# Ophthalmic Complications, Diagnosis, and Treatment of Congenital Human Cytomegalovirus Infection

**DOI:** 10.3390/jcm13123379

**Published:** 2024-06-08

**Authors:** Monika Modrzejewska, Piotr Połubiński, Oliwia Zdanowska

**Affiliations:** 1 2nd Department of Ophthalmology, Pomeranian Medical University in Szczecin, Powstańców Wielkopolskich 72, 70-111 Szczecin, Poland; 2 Scientific Association of Students 2nd Department of Ophthalmology, Pomeranian Medical University in Szczecin, Powstańców Wielkopolskich 72, 70-111 Szczecin, Poland; 3 University Hospital of Karol Marcinkowski in Zielona Góra, 65-046 Zielona Góra, Poland

**Keywords:** CMV, cCMV, ocular complications, chorioretinitis, pregnancy

## Abstract

**Background:** Human cytomegalovirus (hCMV) is the most common etiological agent of congenital infections seen in newborns. Among the most commonly observed complications in children with congenital human cytomegalovirus infection are those affecting the visual system. Ocular complications of congenital CMV (cCMV) are a topic rarely addressed in the literature, which prompted the authors to update the available knowledge with the latest data. **Methodology:** English-language literature published between April 2000 and November 2023 (PubMed, NIH, Google Scholar) was analyzed for ocular complications of cCMV. The data obtained were categorized according to the ocular area involved and the incidence. A compilation of criteria for the symptomatic form of cCMV was also created. **Results:** The cCMV complications described in the literature affect all parts of the visual system: the anterior segment, the posterior segment, the posterior visual pathways, and the visual cortex. The most commonly described ocular complication of cCMV is choroidal and retinal scarring. **Conclusions:** Ophthalmic complications of cCMV can cause severe visual disturbances. Ophthalmic diagnosis in newborns should include hCMV PCR testing, which has the highest sensitivity and specificity. In the symptomatic form of cCMV, treatment should be instituted according to recommendations. A consensus should be established for screening of primary hCMV infection in pregnant women, the way in which to define the symptomatic form of cCMV, and the appropriateness and standards of treatment for primary hCMV infection in pregnant women.

## 1. Introduction

Human cytomegalovirus (hCMV), which is a member of the herpesvirus family, is a pathogen that is specific only to humans but is widespread globally [1].

In healthy individuals with a properly functioning, mature immune system, primary hCMV infection is usually asymptomatic. Less commonly, it can cause mild illness, such as self-limiting fever or a mononucleosis-like syndrome. A much more serious and often complicated form of hCMV infection occurs in patients with various types of immunodeficiency. These include primary immunodeficiency (PID) [2], severe combined immunodeficiency (SCID) [3,4,5], concurrent human immunodeficiency virus (HIV) infection [6,7,8,9], and acute lymphoblastic leukemia (ALL) [10,11,12]. Patients who have undergone hematopoietic stem cell transplantation (HSCT) [13,14,15,16], received chemotherapy [17], or are premature infants [18] also face heightened risks of developing severe organ-specific forms of cytomegalovirus disease [1]. The significant impact of hCMV infection at the population level is underscored by the potentially severe consequences of multiple organ complications resulting from intrauterine infection [19].

Congenital hCMV (congenital cytomegalovirus infection, or cCMV) is the most commonly diagnosed congenital viral infection worldwide, affecting 0.64% of all live births [20]. Such a high prevalence of cCMV is due to the high seroprevalence among women of childbearing age, which reaches 86% in the global population [21]. Transmission of hCMV to the fetus can result from the mother’s primary infection during pregnancy (transmission risk: 30–40%) [22] or reactivation of a previous infection (transmission risk: 1.9%) [23].

According to the literature, approximately 11% of newborns diagnosed with cCMV develop symptomatic disease, which varies in severity and symptomatology [20]. Its most common permanent complications include sensorineural hearing loss (SNHL); neurological and developmental disorders (microcephaly, motor disorders, cognitive impairment, cerebral palsy); vestibular abnormalities; and ophthalmic disorders: choroidal and retinal scarring, optic nerve atrophy, strabismus, cataracts, corneal scarring, and sclerosis [24,25,26].

Due to its significant potential for causing life-threatening prenatal, perinatal, and postnatal pathogenicity, hCMV has been included in the TORCH acronym (Toxoplasma gondii, Others—Parvovirus B19, HIV, Varicella zoster virus; Rubella, Cytomegalovirus, Herpes simplex virus), which represents diseases that can cause birth defects in the fetus when contracted in utero [27,28].

To date, only one paper summarizing ophthalmic complications of cCMV has been published (PubMed), providing an outline of clinical observations from various centers between 1987 and 2010 [29]. In our study, we reviewed the global literature (using PubMed, NIH, and Google Scholar search engines) to summarize and update the current state of knowledge regarding the most common ocular complications of cCMV.

## 2. Methodology

This article is a review of the English-language global literature published between April 2000 and November 2023, conducted using the PubMed, NIH, and Google Scholar search engines. The search utilized various configurations of the subject headings ‘cytomegalovirus’, ‘CMV’, ‘cCMV’, and the keywords ‘congenital’, ‘ophthalmology’, ‘ophthalmologic’, ‘ocular’, ‘visual’, ‘eye’, ‘manifestation’, ‘symptomatology’, ‘symptoms’, and ‘findings’.

These searches led to the selection of six original papers describing ocular complications of cCMV, which we analyzed and evaluated. The data were organized into a table and discussed based on the affected area of the visual system and the incidence of each complication. 

The paper was then supplemented with a current perspective on the diagnosis, prevention, and treatment of cCMV, compiled from 140 articles found through the aforementioned search engines.

The studies reviewed employed varying definitions of symptomatic cCMV. The criteria used by each author are detailed and presented in table form.

**Figure 1:**
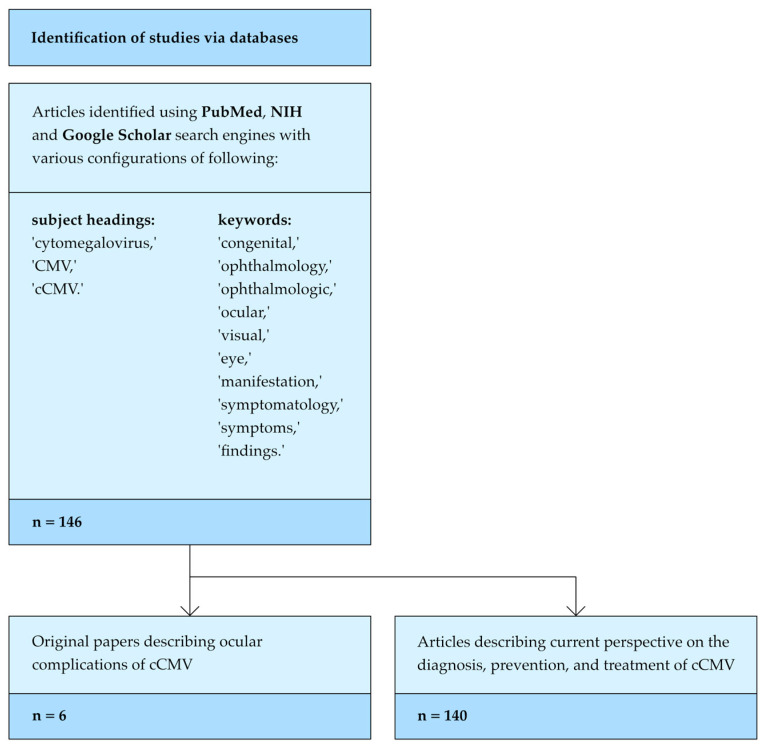
Flow diagram showing the databases used, thematic headers, key words, and the number of selected publications.

The method of identification and selection of articles is presented in Figure 1. 

## 3. Results

### 3.1. Ophthalmic Complications

Ophthalmic complications are among the most commonly observed in the course of cCMV. Approximately 5% to 30% of newborns with cCMV present with ocular lesions [29]. Based on anatomical criteria, complications involving the anterior segment of the eye, the posterior segment of the eye, the visual pathways, and the visual cortex are distinguished [30]. We have presented the compiled data in Table 1. Criteria for symptomatic cCMV as defined by the authors of various studies are presented in Table 2.

#### 3.1.1. Anterior Segment of the Eye

Ocular abnormalities involving the anterior segment of the eye in children with cCMV were described in three of the six case–control studies we analyzed [30,31,32]. Among patients with symptomatic cCMV, isolated cases of punctate superficial keratitis, corneal scarring, reduced corneal dimensions, variegated iris coloration, and anterior polar cataract were observed.

Anterior ocular abnormalities occurring in children with cCMV are among the least frequently observed. Due to their low incidence (similarly low among symptomatic patients, asymptomatic patients, and controls), a direct link between cCMV and the anterior ocular complications described is unlikely. 

#### 3.1.2. Posterior Segment of the Eye

Complications of cCMV involving the posterior segment of the eye occur far more frequently than those affecting the anterior segment and are associated with much more severe visual impairment [30].

Choroidal-retinal scars, which occur in symptomatic patients at a frequency of 5% to 28%, are among the most commonly observed ocular complications of cCMV [30,31,32,33,34,35]. They result from choroidal-retinal inflammation and, depending on their location (peripapillary scars or scars within the macula), can either be asymptomatic or lead to vision loss of varying severity. Choroidal and retinal scars appear as geographic areas of choroidal and retinal atrophy, bordered by regions of hypopigmentation. Their size typically ranges from 0.5 mm to 5 mm [36]. While they are usually inactive, choroidal and retinal scars pose a special diagnostic challenge due to the possibility of late-onset changes within the retina and the potential progression of existing lesions [37].

In addition, isolated cases of retinal vascularization abnormalities related to hCMV infection (occurring without the typical signs of acute choroiditis), similar to those seen in retinopathy of prematurity (ROP), have been reported in the literature [38,39].

Optic nerve atrophy occurs in 4–12% of patients with symptomatic cCMV [30,31,32,33,34] and is one of the most common causes of severe bilateral visual impairment in children diagnosed with cCMV. The exact mechanism leading to optic nerve atrophy in cCMV is unknown. As Coats et al. point out, the atrophy may result from direct infection of the optic nerve’s cellular structures by hCMV during neurogenesis in the first trimester of pregnancy [31].

#### 3.1.3. Posterior Visual Pathways and Visual Cortex

Ophthalmic abnormalities involving the posterior visual pathways and visual cortex in children with cCMV were described in two of the six case–control studies we analyzed [30,31].

In patients with symptomatic cCMV, cerebral visual impairment (CVI) is typically associated with significant vision loss [30]. The incidence of CVI in children with cCMV is approximately 10–14% [30,31]. Children with CVI due to cCMV are also more likely to exhibit other ocular abnormalities compared to those without CVI. This complexity can complicate the diagnosis of CVI and the evaluation of treatment and rehabilitation in this patient group [30]. Similar to optic nerve atrophy resulting from cCMV, the cause of CVI can be attributed to fetal hCMV neuroinfection early in pregnancy [30,31].

#### 3.1.4. Other Ophthalmic Disorders

Other ophthalmic disorders observed in patients with cCMV include strabismus (1.6–29%), amblyopia (4%), astigmatism (29%), and nystagmus (14–15%) [30,31,32,33,34,35].

### 3.2. Diagnostics of cCMV 

**Figure 2:**
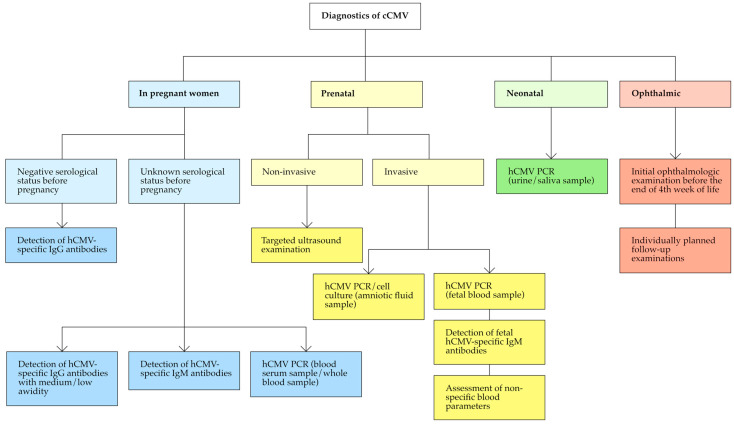
Methods used in the diagnosis of cCMV. cCMV—congenital human cytomegalovirus; hCMV—human cytomegalovirus; IgG—immunoglobulin G; IgM—immunoglobulin M; PCR—polymerase chain reaction.

The methods used in the diagnosis of cCMV are presented in Figure 2.

#### 3.2.1. Diagnostics in Pregnant Women

The goal of diagnostics for hCMV in pregnant women is to detect a primary infection associated with a high risk of hCMV transmission to the fetus [40]. When results of tests performed before pregnancy are available (confirming the pregnant woman’s previously seronegative status), diagnosis is directed at detecting the resulting de novo cytomegalovirus class G (IgG)-specific immunoglobulins present in blood serum. In the case of unknown serological status of the patient before pregnancy, the diagnosis of primary hCMV infection is made on the basis of the detection of hCMV-specific IgG with medium or low avidity, as well as hCMV-specific immunoglobulins in class M (IgM) in the blood serum [40,41,42,43].

Isolated detection of hCMV IgM has a low positive predictive value [44]. Therefore, when early primary hCMV infection is suspected in a pregnant woman (before seroconversion and the appearance of testable IgG levels), polymerase chain reaction (PCR)-based testing using a serum blood sample, or preferentially whole blood, can be performed to confirm primary infection. Up to day 15 of primary infection, hCMV PCR has a positive predictive value of 89% and 100% for tests performed on serum and whole blood samples, respectively [45].

Currently, worldwide, with few exceptions (Israel, Belgium, Canada, and some centers in Italy and France), there is no widespread screening of pregnant women for primary hCMV infection [46]. Serologic diagnosis of hCMV infection (hCMV-specific IgM, IgG, and IgG avidity determination) is recommended primarily for pregnant women with abnormalities found in follow-up imaging studies (magnetic resonance imaging (MRI), ultrasound) and who have experienced flu-like symptoms (fatigue, headache, fever) without any other identifiable cause [41].

#### 3.2.2. Prenatal Diagnosis

A breakdown of the diagnostic methods used to detect hCMV infection in the fetus can be made based on their invasiveness.

An example of a non-invasive diagnostic method is targeted ultrasound. The usefulness of ultrasound in the diagnosis of cCMV stems from its ability to detect and evaluate fetal abnormalities that are potentially the result of intrauterine hCMV infection. The most commonly observed abnormalities include polyhydria, thrombocytopenia, generalized fetal edema, intrauterine fetal growth restriction, placental enlargement, hyperechoic bowel, ventricular dilatation, increased periventricular echogenicity, periventricular pseudocysts, intraventricular adhesions, and cerebral calcifications [47,48,49]. In an affected fetus, more than one abnormality on ultrasound imaging is most often observed simultaneously [49].

Despite its many advantages, including low cost and high availability, the role of ultrasound in diagnosing cCMV during pregnancy is limited. This is mainly due to the low sensitivity and specificity of ultrasound in detecting intrauterine infections.

Abnormalities visualized by ultrasound may also occur as complications of infections of other etiologies [50]. Moreover, defects that may indicate intrauterine hCMV infection are observed on ultrasound in less than half of cCMV cases [51]. Nevertheless, due to the lack of widespread screening, linking abnormalities observed on routine ultrasound to intrauterine hCMV infection often represents the only chance to diagnose cCMV at the fetal life stage [49].

Invasive diagnosis of cCMV is offered to pregnant women with ultrasound findings of fetal abnormalities that may indicate intrauterine infection. An indication for invasive prenatal diagnosis of cCMV is also the finding of a primary hCMV infection in a pregnant woman [41], which has a high risk of virus transmission to the fetus (30–40%) [22].

Invasive prenatal diagnosis of cCMV involves analyzing laboratory samples, such as amniotic fluid collected through amniocentesis and fetal blood obtained via cordocentesis. Amniotic fluid is tested using PCR or cell culture. Fetal blood can also be tested for the presence of hCMV genetic material using PCR. Additionally, fetal IgM titers against hCMV can be measured, along with non-specific, non-viral laboratory parameters of hCMV infection, which include platelet count, hematocrit, hemoglobin concentration, alanine aminotransferase, aspartate aminotransferase, γ-glutamyltransferase, and bound bilirubin levels [52,53,54,55]. Molecular testing of amniotic fluid and fetal blood using PCR has the highest sensitivity (72−100% and 67−93%, respectively). These tests also exhibit high specificity (83−100% and 86−100%, respectively) [53,54,55,56,57,58]. According to the findings of the 2015 International Congenital Cytomegalovirus Recommendations Group, the preferred diagnostic method is quantitative real-time PCR examination of amniotic fluid, performed around 20 weeks into pregnancy and 6–7 weeks after seroconversion indicative of primary maternal infection [41,53].

#### 3.2.3. Neonatal Diagnostics

The diagnostic methods with the highest value for diagnosing cCMV in newborns are urine and saliva samples analyzed using hCMV PCR [41]. Saliva testing has a specificity of 99% and sensitivity of 97% [59], while urine testing has a specificity of 99% and sensitivity of 100% [60]. In their paper, Yamamoto et al. [61] argue that tests using urine and saliva samples are equally reliable for diagnosing cCMV. However, Exler et al. [62] argue that urine testing is the gold standard due to a lower risk of false-positive results, which can occur from contamination of the newborn’s saliva sample with hCMV genetic material from breast milk or contact with genital tract secretions during natural childbirth.

To avoid misdiagnosing cCMV in cases of postnatal cytomegalovirus infection (pCMV), saliva and urine samples should be collected as soon as possible and no later than the end of the third week of life [25,41,60].

Attempts are being made to use dry blood drop material (analogous to screening for inborn metabolic diseases) for cCMV diagnosis (by hCMV PCR) [41,63,64,65].

#### 3.2.4. Ophthalmic Diagnostics

The initial ophthalmologic examination in children with cCMV should be conducted before they reach 4 weeks of age. This facilitates the detection of potential ophthalmic changes due to symptomatic cCMV and informs decisions regarding potential antiviral treatment [35].

The frequency of follow-up examinations and the duration of observation should be tailored individually, based on the patient’s condition and the nature of abnormalities detected in the visual system. It is important to consider that ophthalmic abnormalities and other complications of cCMV, such as hearing impairment [41], may appear later and not be evident during the initial examination [66].

### 3.3. Prevention and Treatment of cCMV

#### 3.3.1. Primary Prevention of Maternal hCMV Infections during Pregnancy

The primary method of preventing primary maternal hCMV infection, and consequently potential transmission of the infection to the fetus, is through properly conducted epidemiological education of the patient. This approach, with proven efficacy [67,68,69,70,71], should preferably extend to all women of reproductive age and pregnant women, particularly during the early stages of pregnancy.

Previous studies [72,73,74] suggest inadequate availability of medical consultations where the topic of cCMV prevention is addressed. Additionally, there is insufficient training among the obstetric community regarding this issue. Consequently, there is low awareness among pregnant women about cCMV prevention [69,75], which is evident in the discrepancy in education levels about hCMV compared to other infectious diseases like toxoplasmosis and listeriosis, which can also lead to fetal damage [76,77].

Comprehensive education should entail providing pregnant women and those planning pregnancies with information about the risks of cCMV and methods to prevent hCMV infection, especially emphasizing caution in situations that expose them to saliva and urine from children [78,79]. Hygiene measures to minimize the risk of hCMV infection include washing hands after changing a child, avoiding contact with secretions when touching a child’s nose and eyes, using separate cutlery, and refraining from kissing a child on the mouth [69,79].

Despite the priority given to public health efforts in both developing and highly developed countries, a widespread formulation for preventing cCMV has yet to be developed [79,80,81,82]. Currently, preparations based on recombinants of the gB subunit have progressed to Phase II clinical trials [82,83,84]. This challenge primarily stems from the unique nature of hCMV infection. Naturally acquired immunity from hCMV infection is insufficient to prevent reactivation of latent infection (by the same strain) or superinfection (by another strain) [80]. A promising avenue of research involves the development of an effective recombinant preparation containing various viral antigens, capable of eliciting both cellular and humoral immune responses against hCMV [81]. Pass et al. demonstrated a 50% efficacy of the tested preparation with anti-hCMV activity, resulting in a reduced frequency of detected cCMV in the study group [82].

#### 3.3.2. Prevention of hCMV Transmission to the Fetus and Treatment If the Fetus Is Found to Be Infected

To prevent vertical transmission of hCMV infection to the fetus, passive immunization is employed. Pregnant women with confirmed primary hCMV infection receive CMV-specific hyperimmunoglobulin (CMVIG) as off-label therapy. The regimen typically involves administration once every 2 weeks at a dosage of 200 IU/kg body weight [85,86,87,88], or once a month at a dosage of 100 IU/kg body weight [89,90]. However, there is currently no consensus on the efficacy of CMVIG or the preferred treatment regimen using this therapy [85,86,87,88,89,90].

Discrepancies in opinions and treatment outcomes are evident in the literature regarding the efficacy of CMVIG therapy. While Kagan et al. highlight the effectiveness of CMVIG with a dosing interval of every 2 weeks [91], Schirwani-Hartl et al. present contrasting findings [92]. The impact of CMVIG on fetal growth rate, the occurrence of obstetric complications, and the risk of premature termination of pregnancy remains subject to debate [89,93]. Further research is necessary to determine the true effectiveness of CMVIG therapy.

The only current method of preventing vertical transmission of hCMV infection to the fetus, with proven statistical and clinical significance in a randomized placebo-controlled trial [94], is antiviral treatment with valacyclovir. Its efficacy has been demonstrated in retrospective and prospective studies. Early initiation of valacyclovir treatment has been associated with better therapeutic outcomes [95,96,97]. According to the revised protocol of Amir et al., treatment with valacyclovir should commence: up to 8 weeks after infection and before 18 weeks of pregnancy in pregnant women diagnosed with infection during the first trimester, and up to 9 weeks for women whose primary hCMV infection occurred between 14 weeks before and up to 10 weeks after conception. Valacyclovir is administered at a dose of 8 g/day [98].

Additionally, studies have demonstrated that the administration of high doses of valacyclovir in cases of confirmed transmission of infection to the fetus results in a significant reduction in hCMV titers in maternal and fetal blood [99]. This approach is also correlated with a decreased incidence of symptomatic cCMV detected at birth [100] and an enhanced prognosis for fetuses displaying symptoms indicative of moderate hCMV infection during pregnancy [101,102].

#### 3.3.3. Management of cCMV Found in a Newborn Baby

Antiviral drugs, such as orally administered valgancyclovir and intravenous gancyclovir, are utilized to treat newborns diagnosed with cCMV. Studies have demonstrated that the use of antiviral drugs in symptomatic neonates with cCMV is associated with improved neurological development [103] and the inhibition of further progression of sensorineural hearing loss (SNHL) [104,105,106]. Furthermore, statistically significant long-term improvements in hearing have been observed, particularly in children with milder degrees of hearing impairment (mild to moderate) detected at birth [104,106,107,108,109,110,111].

The use of gancyclovir and valgancyclovir has been shown to be similarly effective, with valgancyclovir associated with fewer side effects [112].

The most common complication observed in children treated for cCMV with antiviral drugs is neutropenia [113]. Therefore, it is recommended to regularly monitor neutrophil counts in blood tests during treatment. Leung et al. studied the effects of antiviral treatment in neonates with cCMV (*n* = 342), treated with gancyclovir (*n* = 29), valgancyclovir (*n* = 228), or both drugs (*n* = 85). They found that the incidences of neutropenia were 21%, 17%, and 26% for gancyclovir, valgancyclovir, and combination therapy, respectively. When comparing the risk of complications associated with different therapies, it is important to consider the significant differences in treatment protocols: gancyclovir alone is typically administered for a much shorter duration compared to valgancyclovir or combination therapy. Additionally, due to its potential for intravenous administration, gancyclovir can be initiated earlier in the treatment process [114].

As recommended by Chiopris et al., the suggested treatment regimen for cCMV involves oral valgancyclovir at a dosage of 16 mg/kg/dose, administered twice daily for a duration of 12 months [115].

## 4. Discussion

Neglecting early, comprehensive ophthalmic diagnostics in patients with cCMV, particularly when symptomatic forms of the disease are identified, is a dangerous oversight for health. It has been shown that ophthalmic complications occurring in cCMV, especially those affecting the retina, optic nerve, and visual cortex, can cause severe vision disorders, including blindness [30,31,32,33,34,35]. The lack of appropriate treatment and rehabilitation can also lead to secondary ophthalmic complications of cCMV, such as amblyopia and strabismus [31]. It is crucial to establish the exact nature and frequency of ophthalmic complications in cCMV to develop procedures for early diagnosis. An effective diagnostic protocol is essential for implementing early medical interventions and dedicated developmental and educational support in patients with vision-related complications [30]. Coats et al. emphasize the necessity of ophthalmic screening in children with symptomatic cCMV [31], particularly those diagnosed with SNHL [30,32,33] and those receiving cochlear implants [32]. In a study by Lanzieri et al., 43% of children with cCMV and complicated SNHL were found to have concurrent vision disorders [33]. According to findings from a symposium held during the European Society of Paediatric Infectious Diseases conference in 2015, an ophthalmological examination should be part of the essential diagnostics to determine the extent of the disease and make decisions about potential antiviral treatment. Full consensus was reached on the advisability of incorporating antiviral treatment if symptoms of central nervous system involvement are detected, which also include the occurrence of retinochoroiditis in patients with cCMV [116]. The recommendations of the International Congenital Cytomegalovirus Recommendations Group, based on findings from the 5th International Congenital Cytomegalovirus Conference in 2015, include the necessity of conducting early ophthalmic diagnostics during the treatment of patients with cCMV and subsequent follow-up examinations [41].

The effectiveness of antiviral treatment in preventing the progression of cCMV ophthalmic complications and the occurrence of vision disorders remains a matter of debate. Worldwide literature reports cases of stabilization of local retinal conditions or resolution of choroidal and retinal inflammation after antiviral treatment with gancyclovir or valgancyclovir in infants with cCMV [31,117,118,119,120,121,122,123,124]. However, authors of available studies on cCMV treatment often focus on complications other than ophthalmologic ones, primarily hearing disorders and the effectiveness of antiviral treatment in treating them [125,126,127,128,129,130,131]. Consequently, further studies involving a larger number of patients and employing detailed, targeted ophthalmologic diagnostics are necessary to determine the efficacy of antiviral therapy in the treatment of cCMV.

Understanding the clinical picture of ophthalmic complications of cCMV is also crucial for differential diagnosis of cCMV from other causes of concurrent vision, hearing, and balance disorders [132], primarily Usher syndrome. Despite many similarities in clinical presentation, a properly conducted ophthalmic examination can be instrumental in distinguishing between cCMV and Usher syndrome before the inclusion of electrophysiological and genetic testing in the diagnostic process [32].

The risk of reactivation of inflammation in the retina and choroid in patients with cCMV-related changes in the visual system is unknown. The literature describes isolated cases where active changes were detected in ophthalmic examinations of patients with cCMV postnatally [31,37,117,133,134]. Verifying the hypothesis that a recurrence of activity in the retinal changes in patients with cCMV is complicated by the lack of standards regarding the extent of continued ophthalmic observation [30]. In the largest study of its kind to date, conducted by Salomè et al., which included a group of 123 patients with symptomatic cCMV and at least a 6 year period of ophthalmic observation, no new retinal changes were observed in any of the patients [35]. However, it should be noted that antiviral treatment with ganciclovir or valganciclovir was administered to 15 of the 16 children who showed involvement of the visual system in the initial ophthalmic examination. In a retrospective cohort study by Chien et al. on the Taiwanese population, involving a research group of 5797 patients, a significantly higher occurrence of late-onset corneal inflammation was shown in patients with cCMV (*p* = 0.028) [135]. It is important to emphasize that reactivation of inflammation characterized by retinitis and vitreous body inflammation is observed by authors treating preterm infants for retinopathy of prematurity (ROP) [136,137]. In these cases, hCMV infection is nosocomial and arises in response to the compromised immune defenses of heavily and extremely low birth weight preterm infants. Further research is necessary to confirm or exclude the reactivation of changes in the vitreous body, retina, and vascular membrane in pediatric patients with cCMV.

A consensus on the symptomatic form of cCMV is currently lacking. The utilization of different diagnostic schemes by various authors complicates the comparison of the incidence of individual manifestations and ocular complications of cCMV. Only two out of six analyzed studies permitted the diagnosis of the symptomatic form of cCMV based on isolated abnormalities found during fundus examination. In the remaining studies, the ophthalmologic diagnosis was made only after other signs of cCMV had been identified previously [30,31,32,33]. This diagnostic approach makes it challenging to identify cases of isolated ocular cCMV and may lead to a failure to recognize symptoms of ocular disorders. The absence of appropriate diagnosis hinders treatment and planning of ophthalmic follow-up in such cases. To standardize the results of future studies, it is recommended to reconsider the current perspectives of neonatologists, infectious disease physicians, and ophthalmologists on the symptomatic form of cCMV, taking into account the significance of ocular symptoms, and to establish universal diagnostic criteria.

The validity of screening pregnant women for primary hCMV infection remains a contentious issue, partly due to rapidly changing trends in therapies aimed at preventing transmission of the infection to the fetus [40]. Key variables in justifying the introduction of hCMV screening include the effectiveness of available therapies to prevent fetal transmission and the prevalence of primary infection among pregnant women in a given population [138]. Therefore, it is suggested that the rationale for introducing screening should be determined on a country-by-country basis [42,139], preferably based on the outcomes obtained with the most effective available treatments. An argument for universal hCMV screening is the operation of similar programs targeting congenital diseases, albeit less common than cCMV, which cause varying degrees of disability in children, such as toxoplasmosis, rubella, syphilis, early group B streptococcal infection, Down syndrome, and spina bifida [40,139,140]. There is no consensus in the global literature regarding the cost-effectiveness of hCMV screening in pregnant women [138,141,142,143]. However, as demonstrated by Périllaud-Dubois et al., universal hCMV screening combined with valacyclovir-based treatment, particularly when hCMV infection is detected in the first trimester of pregnancy, could be more cost-effective than currently accepted strategies for preventing cCMV and treating its complications [144]. An argument against the introduction of universal hCMV screening is the potential risk of increasing the rate of pregnancy termination in patients diagnosed with primary hCMV infection who may not have developed cCMV or its complications [145]. However, Yves Ville stresses that denying pregnant women access to hCMV screening on this basis is an expression of paternalism, and participating in screening and deciding to proceed with the results is primarily a matter of autonomous patient decision [139]. According to a study by Beaudoin et al. involving 234 pregnant women, the majority (74.4%) expressed interest in serologic screening for primary hCMV infection after being informed about cCMV by their physician [46]. The study revealed that a significant factor in reducing the incidence of pregnancy termination following a positive serologic test was the physician’s consultation, which included appropriate interpretation of the results and counseling on available methods of further diagnosis [146].

## 5. Conclusions

Establishing standards for ophthalmic diagnostics in patients with cCMV would enable more effective early detection of vision-threatening complications, leading to the implementation of appropriate treatment and rehabilitation. Future research should focus on initiating ophthalmic diagnostics as early as possible and extending the observation period to rule out the occurrence of late-onset cCMV complications. Moreover, introducing diagnostic standards for patients with cCMV would allow for an assessment of the actual frequency of various types of ophthalmic complications of cCMV, as well as the effectiveness of recommended treatment regimens. Research findings should be used as a basis for developing national guidelines and administrative protocols. Currently, there is a lack of consensus regarding screening for primary human cytomegalovirus (hCMV) infection in pregnant women, defining symptomatic forms of cCMV, and the appropriateness and standards of treatment for primary hCMV infection in pregnant women. Newborn diagnostics for cCMV should include hCMV PCR testing, characterized by the highest sensitivity and specificity. Upon confirmation of a symptomatic form of cCMV, treatment in accordance with the recommendations should be initiated.

## Figures and Tables

**Table 1:** Ophthalmic complications of cCMV described in individual studies—table created by the authors.

Authors	Years	Symptomatic ^a^ (n)	Asymptomatic ^b^ (n)	Control Group (n)	Complications	Symptomatic ^a^ (n)	Asymptomatic ^b^ (n)	Control Group (n)
Coats et al. [31]	1982–2000	42	83	21	Retinal scarring	9 (21.4%)	2 (2.4%)	0 (0%)
					Within the macular region			
					Unilateral	3 (7.1%)	2 * (2.4%)	
					Bilateral	0 (0%)	0 (0%)	
					Peripheral			
					Unilateral	3 (7.1%)	0 (0%)	
					Bilateral	3 (7.1%)	0 (0%)	
					Strabismus	12 (28.6%)	1 (1.2%)	1 (4.8%)
					Convergent	7 (16.7%)	0 (0%)	0 (0%)
					Divergent	5 (11.9%)	1 (1.2%)	1 (4.8%)
					Optic nerve atrophy	3 (7.1%)	0 (0%)	0 (0%)
					Unilateral	0 (0%)		
					Bilateral	3 (7.1%)		
					Lesions of the anterior segment of the eye	1 (2.4%)	1 (1.2%)	0 (0%)
					Anterior polar cataract	1 (2.4%)	0 (0%)	
					Scarring of the corneal stroma	0 (0%)	1 (1.2%)	
					Cortical visual impairment	4 (9.5%)	0 (0%)	0 (0%)
Teär Fahnehjelm et al. [32]	2002–2012	26	none	13	Choroidal and retinal scars	5 (19.2%)	–	0 (0%)
					Within the macular region			
					Unilateral	5 (19.2%)		
					Bilateral	0 (0%)		
					Peripheral	0 (0%)		
					Strabismus	5 (19.2%)	–	1 (7.7%)
					Optic nerve atrophy	1 (3.8%)	–	0 (0%)
					Lesions of the anterior segment of the eye	1 (3.8%)	–	2 (15.4%)
					Lens opacity	1 (3.8%)		1 (7.7%)
					Corneal erosion	0 (0%)		1 (7.7%)
Lanzieri et al. [33]	1983–2005	76	none	none	Choroiditis and retinitis	19 (25%)	–	–
					Strabismus	19 (25%)	–	–
					Optic nerve atrophy	9 (11.8%)	–	–
					Amblyopia	3 (3.9%)	–	–
					Nystagmus	11 (14.5%)	–	–
					Astigmatism	22 (28.9%)	–	–
Capretti et al. [34]	2006–2015	18	30	none	Retinal scars	5 (27.8%)	0 (0%)	–
					Within the macular region			
					Unilateral	2 (11.1%)		
					Bilateral	0 (0%)		
					Peripheral			
					Unilateral	3 (16.7%)		
					Bilateral	0 (0%)		
					Strabismus	1 (5.6%)	0 (0%)	–
					Convergent	0 (0%)		
					Divergent	1 (5.6%)		
					Optic nerve atrophy	2 (11.1%)	0 (0%)	–
					Unilateral	1 (5.6%)		
					Bilateral	1 (5.6%)		
					Bilateral microphthalmia	1 (5.6%)	0 (0%)	–
Jin et al. [30]	1982–2013	77	109	51	Retinal lesions **	20 (26.0%)	4 (3.7%)	1 (2.0%)
					Unilateral	11 (14.3%)	3 (2.8%)	1 (2.0%)
					Bilateral	8 (10.4%)	0 (0%)	0 (0%)
					Not reported	1 (1.3%)	1 (0.9%)	0 (0%)
					Strabismus	18 (23.4%)	2 (1.8%)	2 (3.9%)
					Convergent	3 (3.9%)	0 (0%)	2 (3.9%)
					Divergent	15 (19.5%)	2 (1.8%)	0 (0%)
					Optic nerve atrophy	8 (10.4%)	0 (0%)	1 (2.0%)
					Unilateral	3 (3.9%)		0 (0%)
					Bilateral	5 (6.5%)		1 (2.0%)
					Amblyopia	3 (3.9%)	2 (1.8%)	2 (3.9%)
					Lesions of the anterior segment of the eye	5 (6.6%)	3 (2.8%)	2 (3.9%)
					Cortical visual impairment	11 (14.3%)	0 (0%)	0 (0%)
					Nystagmus	11 (14.3%)	0 (0%)	0 (0%)
					Astigmatism	22 (28.6%)	18 (16.5%)	9 (17.6%)
Salomè et al. [35]	2002–2022	123	127	none	Choroiditis and retinitis	10 (8.1%)	0 (0%)	–
					Unilateral	4 (3.3%)		
					Bilateral	6 (4.9%)		
					Choroidal and retinal scars	6 (4.9%)	0 (0%)	–
					Unilateral	6 (4.9%)		
					Bilateral	0 (0%)		
					Strabismus	2 (1.6%)	0 (0%)	–
					Convergent	0 (0%)		
					Divergent	2 (1.6%)		
					Abnormalities in the VEP test	8 (6.5%)	0 (0%)	–

cCMV—congenital cytomegalovirus infection, VEP—visual evoked potential; ^a^ patients diagnosed with cCMV described as symptomatic according to criteria adopted by the authors; ^b^ patients with a diagnosis of cCMV described as asymptomatic according to criteria adopted by the authors; * one patient with a periorbital scar; ** retinal lesions including acute choroiditis, retinal scarring, all retinal lesions unrelated to hCMV infection.

**Table 2:** Criteria for the symptomatic form of cCMV—table created by the authors.

Authors	Criteria ^a^
Coats et al. [31]	Presence of one or more of the following symptoms at birth, after prior exclusion of causes other than cCMV: SGA, microcephaly, hepatosplenomegaly, jaundice, thrombocytopenia, petechial rash
Teär Fahnehjelm et al. [32]	Onset of severe SNHL after cCMV requiring treatment with cochlear implants ^b^
Lanzieri et al. [33]	Presence of one or more of the following at birth: microcephaly, hepatosplenomegaly, jaundice, thrombocytopeniac ^c^, petechial rash, unexplained neurological abnormalities, elevated liver enzymes ^d^, hyperbilirubinemiae ^e^, hemolytic anemia
Capretti et al. [34]	Finding: IUGR, hepatomegaly, splenomegaly, petechial rash, thrombocytopenia, elevated plasma transaminases, jaundice ^f^, CNS involvement ^g^
Jin et al. [30]	Presence of one or more of the following symptoms at birth, after prior exclusion of causes other than cCMV: SGA, generalized petechial rash, hepatomegaly, splenomegaly, jaundice, microcephaly, seizures, thrombocytopenia
Salomè et al. [35]	Presence of one or more, of the following symptoms after exclusion of infectious causes other than cCMV: IUGR, splenomegaly, hepatomegaly, thrombocytopenia ^h^, petechial rash, elevated plasma transaminase levels, jaundice ^f^, CNS involvement ^i^

SGA—small for gestational age, SNHL—sensorineural hearing loss, cCMV—congenital cytomegalovirus infection, IUGR—intrauterine growth restriction, CNS—central nervous system, cUS—cranial ultrasound, MRI—magnetic resonance imaging, CT—computed tomography. ^a^ Criteria adopted by the authors and described according to which a symptomatic course of cCMV was found; ^b^ In the admitted group (n = 26), an additional six children were singled out for cCMV symptoms defined as prematurity, jaundice, petechial rash, hepatosplenomegaly; ^c^ Platelets < 75,000/mm^3^; ^d^ Alanine aminotransferase > 100 IU; ^e^ Total bilirubin > 3 mg/dL; ^f^ Elevated levels of conjugated bilirubin; ^g^ Microcephaly, seizures, abnormalities on neuroimaging studies (cUS, MRI), SNHL, abnormalities found in fundus examination; ^h^ Platelets < 100,000/mm^3^; ^i^ Microcephaly, seizures, sucking disorders, lethargy, abnormalities on neuroimaging studies (cUS, MRI, CT), SNHL, abnormalities found in fundus examination.

## Data Availability

No new data were created or analyzed in this review. Data sharing does not apply to this article.
